# Stability of HIV-1 Nucleic Acids in cobas Plasma Separation Card for Viral Load Measurement

**DOI:** 10.1093/ajcp/aqac007

**Published:** 2022-02-05

**Authors:** Adolfo Vubil, Carina Nhachigule, Ana Flora Zicai, Bindiya Meggi, Paulino da Costa, Nédio Mabunda, Sofia Viegas, Nádia Sitoe, Ilesh Jani

**Affiliations:** Instituto Nacional de Saúde, Marracuene, Mozambique; Instituto Nacional de Saúde, Marracuene, Mozambique; Instituto Nacional de Saúde, Marracuene, Mozambique; Instituto Nacional de Saúde, Marracuene, Mozambique; Instituto Nacional de Saúde, Marracuene, Mozambique; Instituto Nacional de Saúde, Marracuene, Mozambique; Instituto Nacional de Saúde, Marracuene, Mozambique; Instituto Nacional de Saúde, Marracuene, Mozambique; Instituto Nacional de Saúde, Marracuene, Mozambique

**Keywords:** HIV viral load, Nucleic acids, Stability

## Abstract

**Objectives:**

Our study aimed to evaluate the stability of human immunodeficiency virus 1 (HIV-1) RNA on cobas plasma separation card (PSC) specimens for viral load (VL) testing after being exposed to varied temperatures and storage times.

**Methods:**

For this purpose, venous PSC specimens were collected and stored at 25ºC to 42ºC for a period of up to 28 days. Plasma VL at baseline was used as reference, against which PSC VL was compared at different time points.

**Results:**

From the 30 patients included in the study, 600 PSC and 30 fresh plasma specimens were obtained. Plasma VL at baseline was fewer than 1,000 copies/mL in 16 patients, and 99.4% of PSCs from these patients yielded nonquantifiable VL at all temperature ranges and time points. During the study period, minor variation of VL was observed in PSCs obtained from 13 patients with plasma VL fewer than 1,000 copies/mL at baseline. For the patient with plasma VL at 1,000 copies/mL, the PSC VL varied from undetectable to 1,670 copies/mL.

**Conclusions:**

Our results show minor variation of VL in PSC specimens in the study conditions. HIV RNA is stable in PSCs exposed to high temperatures for up to 28 days.

**KEY POINTS**

- The stability of human immunodeficiency virus 1 (HIV-1) RNA for viral load testing on cobas plasma separation card (PSC) specimens was evaluated after exposure to various temperatures and storage times.
- HIV-1 RNA is stable in cobas PSC specimens at up to 42°C during 28 days.
- The cobas PSC specimen is a viable alternative to fresh plasma for HIV viral load determination in hard-to-reach settings.

## INTRODUCTION

Monitoring of human immunodeficiency virus 1 (HIV-1) viral load (VL) during antiretroviral treatment is best achieved in fresh plasma. However, the use of this specimen type has been hampered in sub-Saharan Africa due to (1) the scarcity of well-equipped laboratories, which exist only in a few main urban centers,^1,2^ and (2) challenges for the fast transportation of specimens from hundreds of primary health care facilities to the testing laboratories.^3^ Dried blood spots (DBSs) have been widely implemented as a viable alternative specimen for HIV-1 VL measurement.^4-8^ Advantages of DBSs include the possibility of transportation under easier biosafety conditions at room temperature. However, due to the interference of proviral DNA and intracellular RNA, VL values determined using DBSs are known to be overestimated at the clinically relevant range of 1,000 copies/mL.^7,9-11^

Recently, the cobas plasma separation card (PSC) was shown to be a more accurate alternative to DBSs.^12,13^ Despite this recent evidence of the good performance of the PSC for HIV-1 VL testing, it is unknown whether HIV-1 RNA in the PSC remains stable when exposed to higher temperatures in the tropics. These data are important for the establishment of functional referral systems in sub-Saharan Africa, given the variability of temperature during transportation from clinics to the testing laboratory. Our study aimed at evaluating the stability of HIV-1 RNA for VL testing on the cobas PSC after exposure to various temperatures and storage times.

## MATERIALS AND METHODS

### Study Design and Participants

HIV-infected adults initiating antiretroviral therapy (ART) in Primeiro de Maio Health Center in Maputo City were consecutively enrolled in the study in July 2019. Beside the DBS specimens routinely collected for HIV-1 VL monitoring, additional venous blood was collected to prepare the cobas PSC specimens and fresh plasma.

### Specimen Collection and Preparation

The cobas PSC (Roche Molecular Systems) is a novel device that allows whole blood collection and filtration into dried plasma spots. The device comprises a porous membrane, which retains some blood components and allows plasma to filter through and be collected onto an underlying polyester fleece. The latter is impregnated with an RNA stabilizing reagent. After collection and drying, the specimen on the card can be packed and transported to a laboratory for HIV-1 VL testing.^12,13^

For each patient, 12 mL of venous blood was collected in a BD Vacutainer K_2_EDTA and sent to the Instituto Nacional de Saúde laboratory within 6 hours after venesection, where 20 cobas PSC specimens were prepared for each patient. The remnant whole blood was immediately centrifuged at 800 to 1,600*g* for 20 minutes at room temperature to obtain plasma, which was stored at –80ºC until testing.

For the PSC card preparation, 140 μL of whole blood was transferred onto each of the three delineated areas of the card and dried overnight at room temperature. After drying, cards were immediately packed in airtight plastic bags containing desiccant gel and humidity indicators and stored during 1, 3, 7, 21, and 28 days under four time range–controlled temperatures of 2°C to 8°C, 25°C, 37°C, and 42°C up to the testing day. Whenever the color of the humidity indicator changed from blue to pink, both the humidity indicator and desiccant gel were replaced with new ones.

### VL Testing

For both plasma and PSC specimens, VL was tested using the Roche CAP/CTM 96 HIV-1 Quantitative Test v2 (Roche Molecular Diagnostics), according to the manufacturer’s instructions. Interpretation of VL results was performed according to the manufacturer’s instructions, which establish 20 and 738 copies/mL of viral RNA as the low limit of quantification (LoQ) for fresh plasma and cobas PSC specimens, respectively.

### Statistical Analysis

All VL results were transformed to log_10_ copies/mL. Specimens with nondetectable VL results and with values below the LoQ, for both plasma and PSC specimens, were assigned a value of 1 copy/mL to enable quantitative log_10_ copies/mL transformation. Specimen types were categorized by plasma VL results and based on the LoQ of the cobas PSC (738 copies/mL). The stability of nonquantifiable results is reported as percent concordance. The absolute VL result is provided where discordance is noted. The fresh plasma VL result (24-hour specimen) was taken as the reference against which all PSC VL results were compared over time and by temperature category. Bland-Altman analysis was used to measure the mean difference and limits of agreement between plasma VL at baseline and cobas PSC VL for different storage temperatures and time points.^14^ A difference of less than 0.3 log copies/mL was considered acceptable and had no implications for clinical follow-up.^15^

### Ethical Considerations

Ethical approval for the study was obtained from Mozambique’s National Health Bioethics Committee (reference number 297/CNBS). Written informed consent was obtained from each patient prior to conducting any study procedure.

## RESULTS

Thirty HIV-infected adults initiating ART schemes were enrolled for the study in July 2019. The median age of patients was 39 years, and most (56.7%) were female.

Of 30 patients included in the study, 16 had plasma VL fewer than 1,000 copies/mL: 3 had undetectable plasma VL, 6 had VL fewer than 20 copies/mL, and 7 had VL fewer than 738 copies/mL. One patient had a VL at 1,000 copies/mL, and 13 patients had a VL between 3,000 and 800,000 copies/mL. The 600 PSC cards that were generated from the 30 patients recruited for this study were exposed to various storage temperatures and times **TABLE 1**.

**TABLE 1:** Mean Difference of Viral Load Results Over the 28-Day Study Period in Temperature Range of 25ºC to 42ºC

Plasma VL Category	n	24 Hours	72 Hours	7 Days	21 Days	28 Days
2°C-8ºC PSC specimens						
Target not detected, %	3	100	100	100	100	100
Target <20 cp/mL, %	6	100	100	100	100	100
Target quantifiable but <738 cp/mL, %	7	100	100	100	100	100
Target at 1,000 cp/mL, c/mL	1	1,486	TND	<738	TND	820
>1,000 cp/mL (range, 3,000-800,000 cp/mL), mean (SD) difference	13	–0.31 (–0.09)	–0.30 (–0.15)	–0.29 (–0.04)	–0.22 (–0.09)	–0.25 (–0.12)
25ºC PSC specimens						
Target not detected, %	3	100	100	100	100	100
Target <20 cp/mL, %	6	100	100	100	100	100
Target quantifiable but <738 cp/mL, %	7	100	100	100	100	100
Target at 1,000 cp/mL, c/mL	1	949	995	<738	771	<738
>1,000 cp/mL (range, 3,000-800,000 cp/mL), mean (SD) difference	13	–0.31 (–0.17)	–0.30 (–0.09)	–0.29 (–0.13)	–0.22 (–0.17)	–0.25 (–0.10)
37ºC PSC specimens						
Target not detected, %	3	100	100	100	100	100
Target <20 cp/mL, %	6	100	100	100	100	100
Target quantifiable but <738 cp/mL, %	7	100	100	100	86^a^	86^a^
Target at 1,000 cp/mL,^a^ c/mL	1	1,087	TND	<738	749	TND
>1,000 cp/mL (range, 3,000-800,000 cp/mL), mean (SD) difference	13	–0.28 (–0.10)	–0.25 (–0.11)	–0.22 (–0.23)	–0.05 (–0.28)	–0.07 (–0.19)
42ºC PSC specimens						
Target not detected, %	3	100	100	100	100	100
Target <20 cp/mL, %	6	100	100	100	100	100
Target quantifiable but <738 cp/mL, %	7	100	100	100	100	100
Target at 1,000 cp/mL, c/mL	1	<738	TND	TND	936	1,670
>1,000 cp/mL (range, 3,000-800,000 cp/mL), mean (SD) difference	13	–0.28 (–0.06)	–0.28 (–0.10)	–0.28 (–0.06)	–0.20 (–0.07)	–0.05 (–0.17)

cp/mL, copies per milliliter; PSC, plasma separation card; VL, viral load.

^a^n = 1 at 1,354 cp/mL; n = 1 at 2,954 cp/mL.

Almost all of the PSC cards (318/320; 99.4%) from the 16 patients with plasma VL fewer than 1,000 copies/mL at the baseline remained with nonquantifiable VL results at all temperature ranges and time points over the 28 days of the study. A minor proportion of these PSC specimens (2/320; 0.6%) generated quantifiable VL values of 1,354 copies/mL and 2,954 copies/mL in two cards obtained from two different patients, which were stored at 37ºC during 21 and 28 days, respectively.

The quantification of viral RNA for the patient with plasma VL at 1,000 copies/mL yielded results ranging between target not detected and 1,670 copies/mL TABLE 1. The best results for these specimens were yielded at 25ºC.

**FIGURE 1:**
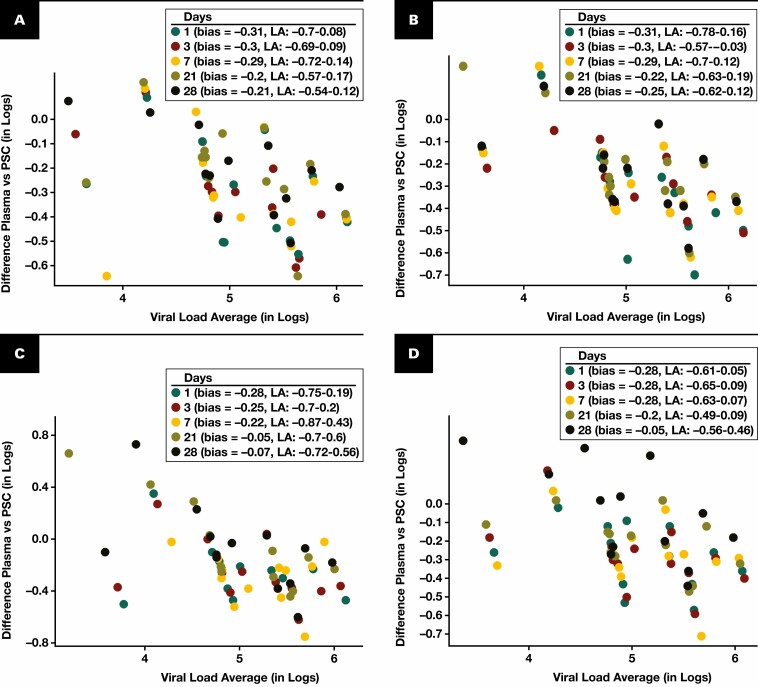
Bland-Altman analyses to evaluate agreement in human immunodeficiency virus 1 viral load quantification among plasma and cobas plasma separation card (PSC) specimens stored at temperatures ranging from 2ºC to 8ºC (**A**), 25ºC (**B**), 37ºC (**C**), and 42ºC (**D**) for a period of up to 28 days. LA, limit of agreement.

A total of 260 PSC cards were generated from 13 patients with plasma VL between 3,000 and 800,000 copies/mL TABLE 1**FIGURE 1**. In general, PSC specimens showed a slight reduction in viral nucleic acid quantities after storage for up to 28 days. Specimens stored at 2ºC to 8ºC FIGURE 1A showed a mean difference of –0.31, –0.30, –0.29, –0.20, and –0.21 log copies/mL when stored for 1, 3, 7, 21, and 28 days, respectively. Similar minor mean differences were observed in PSC specimens stored at 25ºC FIGURE 1B, 37ºC FIGURE 1C, and 42ºC FIGURE 1D.

All specimens from the 13 patients with VL values more than 3,000 copies/mL had a variance less than 0.3 log copies/mL at all time points and temperatures of the study, except at day 1 at a temperature of 2ºC to 8ºC and 25ºC, in which the variance was 0.31 log copies/mL.

The mean differences displayed for the various temperature and time points of the study are in line with the tolerable difference of less than 0.3 log copies/mL and should not be of clinical significance for the follow-up of patients.^15^

## DISCUSSION

The use of plasma for measuring HIV-1 VL in resource-limited settings poses formidable logistical challenges. Although the introduction of DBSs has contributed to increased access to HIV-1 VL testing, this specimen type yields inaccurate results due to the presence of proviral DNA and RBC-associated viral RNA. The novel PSC retains both the logistical advantages of DBS specimens and the accuracy of plasma for HIV-1 RNA measurement.

Viral loads determined on PSC specimens have been shown to be in agreement with those measured in fresh plasma. A study conducted in South Africa to seek the performance of PSCs to identify virologic failure at the threshold of 1,000 copies/mL yielded a sensitivity of 97.0% (92.4%-99.2%) and a specificity of 97.2% (94.9%-98.6%). The misclassification rate in this study was 2.9%.^13^ Additional data from a similar study recently conducted in Mozambique yielded a sensitivity of 99.8% (99.8%-100%) and 100% (99.2%-100%) in capillary and venous PSC specimens, respectively. The specificity was 97.3% (92.4%-99.4%) in capillary PSC specimens and 98.2% (93.6%-99.8%) in venous PSC specimens. The misclassification rate for this study was 0.7% in capillary PSC specimens and 0.3% in venous PSC specimens.^12^

Our results further show that plasma HIV-1 VLs are in close agreement with those generated from PSC specimens stored for up to 28 days after collection. Moreover, PSC storage at temperatures as high as 42ºC does not negatively affect the accuracy of HIV-1 VL. The discrepant results observed in two cards that showed nonquantifiable VL at baseline but generated quantifiable VL results when stored at 37ºC during 21 and 28 days, respectively, may be due to deficiency on the porous membrane of those specific cards, which compromised the retention of blood components during specimen preparation. This could cause incorrect categorization of viral suppression and potentially lead to an unnecessary switch of the ARV scheme. However, general data from our study showed a minor decrease of the VL values, with no clinical significance across all storage temperatures and time points of the study.^15^ Our findings are in line with results from a previous study that showed that the cobas PSC specimen can be stored for 56 days between 2ºC and 30ºC, without significant variance on the HIV-1 VL result.^13^

Humidity is another factor that can influence stability of biological specimens. However, humidity conditions were not evaluated in the present study. As a standard procedure in sub-Saharan Africa, DBS and PSC cards are stored and transported in airtight plastic bags containing desiccant gel and humidity indicators. This practice has been adopted to mitigate the negative effects of the humidity.

It is also important to note that the higher LoQ of the cobas PSC specimen (738 copies/mL) compared with fresh plasma (20 copies/mL) requires more frequent VL monitoring at 3-month intervals, especially for patients with VL lower than 1,000 copies/mL. Nevertheless, the cobas PSC specimen has been shown to be suitable to identify virologic failure and treatment success at this clinically relevant threshold.^12^ The stability of PSC specimens stored at high temperatures for up to 28 days is an important feature for field conditions in resource-limited settings, given that in most sub-Saharan African countries, preshipment storage at health facilities and transportation of specimens to the reference laboratories can take up to a month.^16^

## CONCLUSIONS

In addition to the good performance for VL quantification reported in previous studies, this study demonstrated that HIV VL can be accurately determined in PSC cards stored at temperatures as high as 42ºC for up to 28 days. Taken together, these data support the use of the cobas PSC as a good alternative to fresh plasma for the laboratory monitoring of ART in resource-limited settings.

## Notes

Funding: The Bill and Melinda Gates Foundation (grant No. OPP1214435).
